# Crystal structure of 3,9,9-trimethyl-2,3,3a,4,9,9a-hexa­hydro-1*H*-cyclo­penta[*b*]quinolin-4-ium chloride

**DOI:** 10.1107/S2056989015011858

**Published:** 2015-06-27

**Authors:** G. Sridhar, I. Mohammed Bilal, D. Easwaramoorthy, S. Kutti Rani, K. Anand Solomon

**Affiliations:** a Department of Chemistry, B.S. Abdur Rahman University, Chennai 600 048, India; b Department of Chemistry, Sri Sathya Sai Center for Human Excellence, Karnataka 562 101, India

**Keywords:** crystal structure, quinoline, N—H⋯Cl hydrogen bonds

## Abstract

The title mol­ecular salt, C_15_H_22_N^+^·Cl^−^, arose as an unexpected product of the reaction between aniline and melanol in the presence of HCl. The central heterocyclic ring has a half-chair conformation and the five-membered ring has an envelope conformation, with the C atom linked to the N atom as the flap. In the crystal, the ions are linked by N—H⋯Cl hydrogen bonds, generating chains propagating in the [100] direction. The crystal studied was a merohedral twin with a 0.64 (3):0.36 (3) domain ratio.

## Related literature   

For biological background, see: Szymański *et al.* (2012 ▸). For further synthetic details, see: Alaghaz *et al.* (2014 ▸).
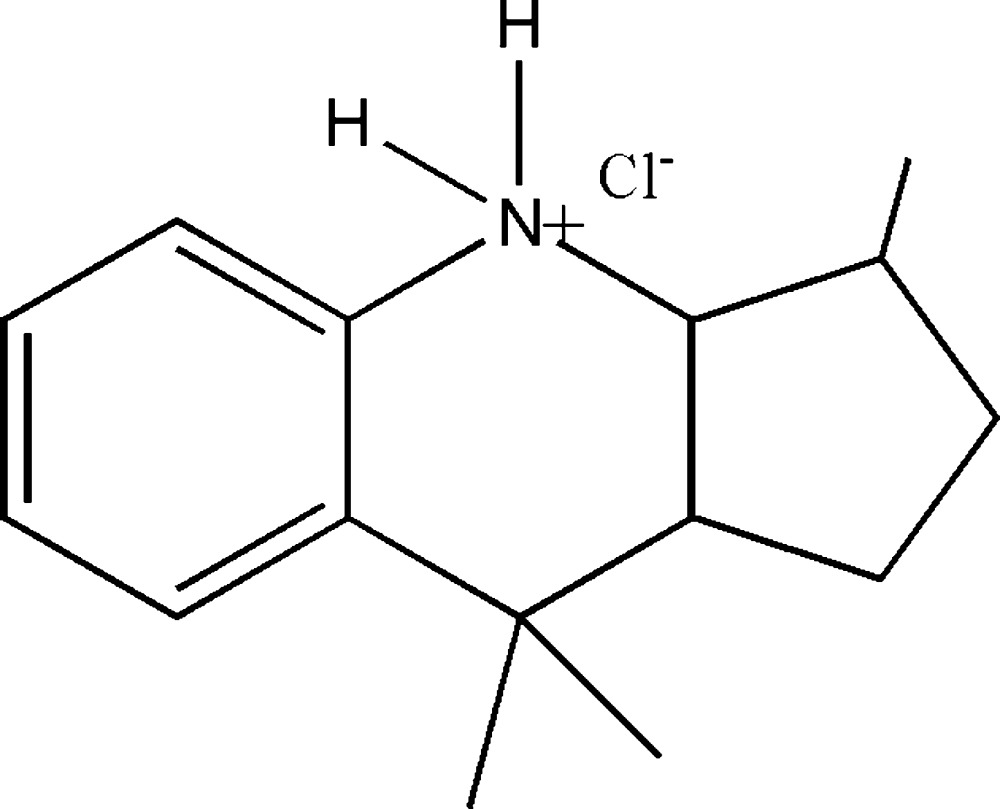


## Experimental   

### Crystal data  

C_15_H_22_N^+^·Cl^−^
*M* *_r_* = 251.78
Orthorhombic, 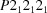

*a* = 7.0291 (5) Å
*b* = 10.3313 (8) Å
*c* = 18.9425 (14) Å
*V* = 1375.60 (18) Å^3^
*Z* = 4
Mo *K*α radiation
μ = 0.26 mm^−1^
*T* = 298 K
0.35 × 0.30 × 0.30 mm

### Data collection  

Oxford Diffraction Xcalibur Sapphire3 diffractometer
7372 measured reflections
3334 independent reflections
3013 reflections with *I* > 2σ(*I*)
*R*_int_ = 0.028
Standard reflections: 0

### Refinement  

*R*[*F* ^2^ > 2σ(*F* ^2^)] = 0.039
*wR*(*F* ^2^) = 0.123
*S* = 0.95
3334 reflections
165 parameters
H atoms treated by a mixture of independent and constrained refinement
Δρ_max_ = 0.26 e Å^−3^
Δρ_min_ = −0.23 e Å^−3^
Absolute structure: Flack *x* determined using 1165 quotients [(*I* ^+^)−(*I* ^−^)]/[(*I* ^+^)+(*I* ^−^)] (Parsons *et al.*, 2013 ▸)
Absolute structure parameter: 0.36 (3)

### 

Data collection: *CrysAlis PRO* (Oxford Diffraction, 2010 ▸); cell refinement: *CrysAlis PRO*; data reduction: *CrysAlis PRO*; program(s) used to solve structure: *SHELXS7* (Sheldrick, 2008 ▸); program(s) used to refine structure: *SHELXL2014*/7 (Sheldrick, 2015 ▸); molecular graphics: *ORTEP-3 for Windows* (Farrugia, 2012 ▸) and *Mercury* (Macrae *et al.*, 2008 ▸); software used to prepare material for publication: *SHELXL97* (Sheldrick, 2008 ▸).

## Supplementary Material

Crystal structure: contains datablock(s) I, New_Global_Publ_Block. DOI: 10.1107/S2056989015011858/hb7450sup1.cif

Structure factors: contains datablock(s) I. DOI: 10.1107/S2056989015011858/hb7450Isup2.hkl

Supporting information file. DOI: 10.1107/S2056989015011858/hb7450Isup3.cml

**Fig. 1.**
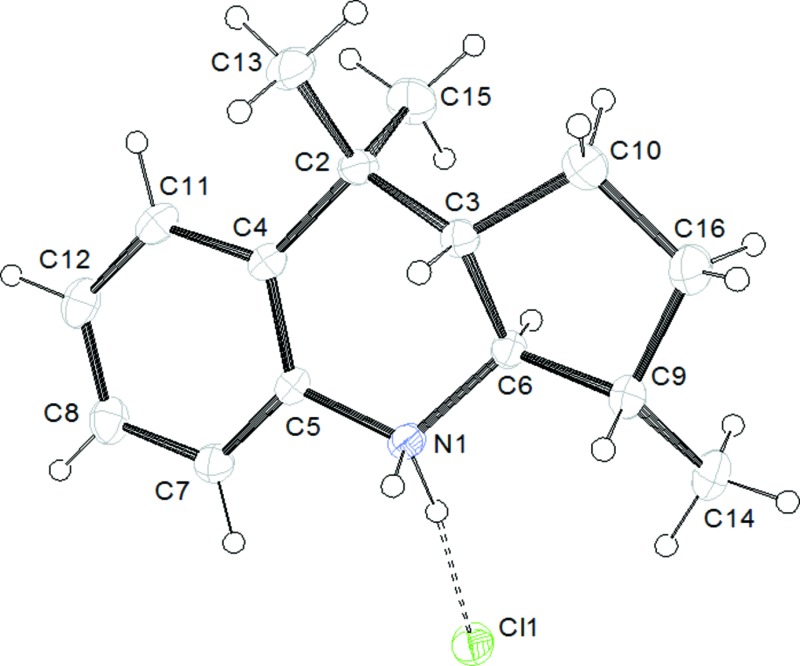
ORTEP diagram of the title compound drawn at 30% probability

ORTEP . DOI: 10.1107/S2056989015011858/hb7450fig1.tif
*ORTEP* diagram of the title compound drawn at 30% probability

**Fig. 2.**
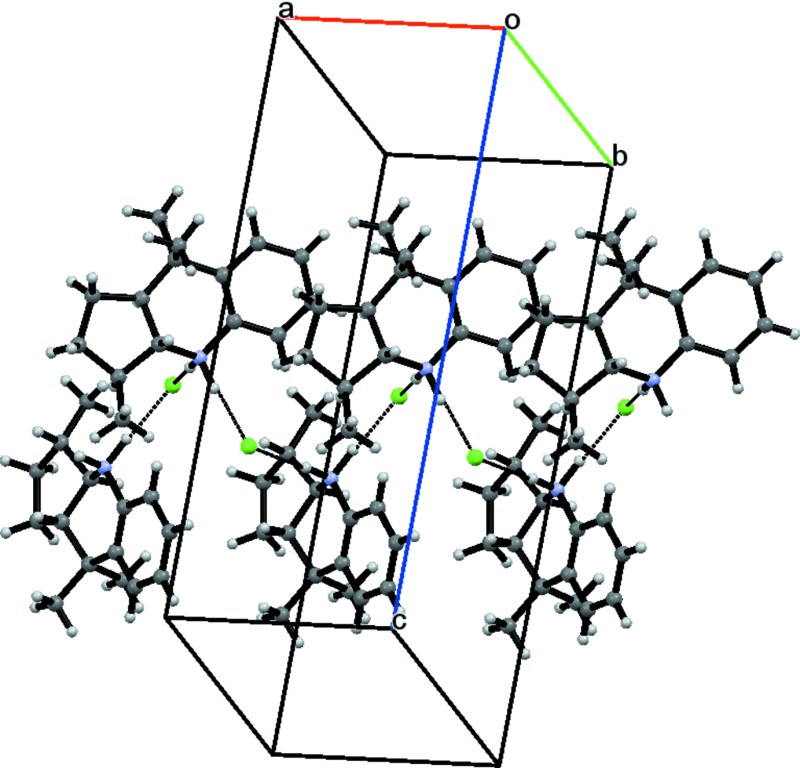
Packing diagram of the molecule viewed down 'b' axis

. DOI: 10.1107/S2056989015011858/hb7450fig2.tif
Packing diagram of the mol­ecule viewed down ’b′ axis

CCDC reference: 1407884

Additional supporting information:  crystallographic information; 3D view; checkCIF report

## Figures and Tables

**Table 1:** Hydrogen-bond geometry (, )

*D*H*A*	*D*H	H*A*	*D* *A*	*D*H*A*
N1H1*A*Cl1	1.05(4)	2.07(4)	3.1201(19)	173(3)
N1H1*B*Cl1^i^	0.93(3)	2.17(3)	3.0943(19)	174(3)

Symmetry code: (i) 
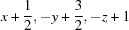
.

## supplementary crystallographic information

### S1. Introduction

Tacrine was the drug approved by the United States Food and Drug Administration in 1993 for the palliative treatment of Alzheimer's Disease. The derivatives of the title compound, which is a congener of tacrine, has also been reported to be effective anti-alzheimeric agents (Szymanski *et al.*, 2012). In this salt the N atom is protonated with sp3 hybridization and with a tetra­hedral geometry. In the crystal, the molecules are linked via N—H···Cl bonds forming chains propagating along the a axis direction.The five membered ring adopts an envelope conformation, while the six-membered non aromatic ring adopts a twist-chair conformation as evinced from Puckering amplitude θ=46.50 (0.15), Ψ= -91.77(0.26), QT=0.5104 (1).

### S2.1. Synthesis and crystallization

The quinoline derivative was prepared by the condensation 2,6-di­methyl-5- heptenaldehyde and aniline in 1:1 molar ratio by refluxing in propan-2-ol using HCl as a catalyst. The mixture was left under reflux for 3 h.

The solution was then left at room temperature. The solid product formed was separated by filtration, purified by crystallized with ethanol and washed with acetone and then dried in a vacuum over anhydrous calcium chloride (Alaghaz *et al.*, 2014). The beige coloured product was formed in 80% yield.

### S2.2. Refinement

The C-bound H atoms were included in calculated positions and treated as riding atoms: C—H = 0.93 Å with Uiso(H) = 1.2Ueq(C).

### Crystal data

**Table:**

C_15_H_22_N^+^·Cl^−^	*D*_x_ = 1.216 Mg m^−^^3^
*M**_r_* = 251.78	Mo *K*α radiation, λ = 0.71073 Å
Orthorhombic, *P*2_1_2_1_2_1_	Cell parameters from 25 reflections
*a* = 7.0291 (5) Å	θ = 2.0–25°
*b* = 10.3313 (8) Å	µ = 0.26 mm^−^^1^
*c* = 18.9425 (14) Å	*T* = 298 K
*V* = 1375.60 (18) Å^3^	Block, colourless
*Z* = 4	0.35 × 0.30 × 0.30 mm
*F*(000) = 544	

### Data collection

**Table:**

Oxford Diffraction Xcalibur Sapphire3 diffractometer	*R*_int_ = 0.028
ω scans	θ_max_ = 28.5°, θ_min_ = 2.2°
7372 measured reflections	*h* = −9→9
3334 independent reflections	*k* = −13→9
3013 reflections with *I* > 2σ(*I*)	*l* = −10→24

### Refinement

**Table:**

Refinement on *F*^2^	Hydrogen site location: mixed
Least-squares matrix: full	H atoms treated by a mixture of independent and constrained refinement
*R*[*F*^2^ > 2σ(*F*^2^)] = 0.039	*w* = 1/[σ^2^(*F*_o_^2^) + (0.1*P*)^2^] where *P* = (*F*_o_^2^ + 2*F*_c_^2^)/3
*wR*(*F*^2^) = 0.123	(Δ/σ)_max_ < 0.001
*S* = 0.95	Δρ_max_ = 0.26 e Å^−^^3^
3334 reflections	Δρ_min_ = −0.23 e Å^−^^3^
165 parameters	Absolute structure: Flack *x* determined using 1165 quotients [(*I*^+^)-(*I*^-^)]/[(*I*^+^)+(*I*^-^)] (Parsons *et al.*, 2013)
0 restraints	Absolute structure parameter: 0.36 (3)

### Special details

**Table:**

Geometry. All e.s.d.'s (except the e.s.d. in the dihedral angle between two l.s. planes) are estimated using the full covariance matrix. The cell e.s.d.'s are taken into account individually in the estimation of e.s.d.'s in distances, angles and torsion angles; correlations between e.s.d.'s in cell parameters are only used when they are defined by crystal symmetry. An approximate (isotropic) treatment of cell e.s.d.'s is used for estimating e.s.d.'s involving l.s. planes.

### Fractional atomic coordinates and isotropic or equivalent isotropic displacement parameters (Å^2^)

**Table:**

	*x*	*y*	*z*	*U*_iso_*/*U*_eq_	
Cl1	0.15461 (9)	0.64944 (6)	0.56931 (3)	0.0429 (2)	
N1	0.4133 (3)	0.60045 (17)	0.43815 (9)	0.0287 (4)	
C2	0.5014 (3)	0.4292 (2)	0.31431 (12)	0.0311 (5)	
C3	0.6364 (3)	0.4629 (2)	0.37502 (12)	0.0306 (4)	
H3	0.6958	0.5458	0.3630	0.037*	
C4	0.3378 (3)	0.5258 (2)	0.31593 (10)	0.0282 (4)	
C5	0.2938 (3)	0.6012 (2)	0.37467 (11)	0.0272 (4)	
C6	0.5333 (3)	0.4825 (2)	0.44441 (11)	0.0284 (4)	
H6	0.4519	0.4074	0.4539	0.034*	
C7	0.1374 (3)	0.6827 (2)	0.37588 (13)	0.0360 (5)	
H7	0.1104	0.7308	0.4162	0.043*	
C8	0.0217 (3)	0.6927 (3)	0.31749 (14)	0.0409 (6)	
H8	−0.0835	0.7473	0.3181	0.049*	
C9	0.6887 (4)	0.4896 (2)	0.49987 (12)	0.0377 (5)	
H9	0.7489	0.5750	0.4975	0.045*	
C10	0.7987 (4)	0.3694 (3)	0.39459 (15)	0.0497 (7)	
H10A	0.7630	0.2806	0.3844	0.060*	
H10B	0.9133	0.3901	0.3684	0.060*	
C11	0.2179 (3)	0.5383 (3)	0.25764 (12)	0.0375 (5)	
H11	0.2427	0.4897	0.2173	0.045*	
C12	0.0635 (3)	0.6210 (3)	0.25812 (14)	0.0420 (6)	
H12	−0.0127	0.6283	0.2182	0.050*	
C13	0.6117 (4)	0.4384 (3)	0.24472 (13)	0.0463 (6)	
H13A	0.6450	0.5271	0.2359	0.070*	
H13B	0.5338	0.4068	0.2068	0.070*	
H13C	0.7254	0.3873	0.2479	0.070*	
C14	0.6223 (4)	0.4657 (3)	0.57507 (13)	0.0518 (7)	
H14A	0.5301	0.5300	0.5879	0.078*	
H14B	0.7291	0.4707	0.6065	0.078*	
H14C	0.5659	0.3813	0.5783	0.078*	
C15	0.4183 (5)	0.2923 (2)	0.32124 (16)	0.0508 (7)	
H15A	0.5201	0.2305	0.3240	0.076*	
H15B	0.3407	0.2736	0.2808	0.076*	
H15C	0.3423	0.2871	0.3632	0.076*	
C16	0.8301 (5)	0.3879 (3)	0.47281 (15)	0.0598 (8)	
H16A	0.9594	0.4165	0.4815	0.072*	
H16B	0.8107	0.3066	0.4974	0.072*	
H1A	0.334 (6)	0.613 (4)	0.4846 (18)	0.079 (11)*	
H1B	0.490 (4)	0.673 (3)	0.4388 (16)	0.047 (8)*	

### Atomic displacement parameters (Å^2^)

**Table:**

	*U*^11^	*U*^22^	*U*^33^	*U*^12^	*U*^13^	*U*^23^
Cl1	0.0449 (3)	0.0503 (3)	0.0335 (3)	0.0088 (3)	0.0067 (2)	0.0008 (3)
N1	0.0305 (8)	0.0319 (8)	0.0238 (9)	0.0006 (7)	−0.0019 (7)	−0.0030 (7)
C2	0.0361 (10)	0.0296 (10)	0.0275 (11)	0.0002 (8)	0.0007 (8)	−0.0042 (8)
C3	0.0310 (10)	0.0334 (10)	0.0275 (11)	0.0030 (8)	0.0009 (8)	−0.0018 (8)
C4	0.0277 (9)	0.0315 (9)	0.0255 (10)	−0.0056 (9)	0.0017 (8)	−0.0010 (8)
C5	0.0273 (9)	0.0319 (9)	0.0223 (10)	−0.0029 (7)	−0.0007 (7)	0.0001 (8)
C6	0.0324 (10)	0.0280 (9)	0.0247 (10)	0.0000 (8)	−0.0022 (7)	0.0014 (8)
C7	0.0354 (11)	0.0402 (11)	0.0324 (11)	0.0052 (9)	0.0016 (9)	−0.0012 (9)
C8	0.0300 (10)	0.0514 (14)	0.0412 (14)	0.0054 (10)	−0.0023 (9)	0.0065 (11)
C9	0.0411 (12)	0.0390 (11)	0.0330 (11)	0.0053 (10)	−0.0102 (9)	−0.0005 (10)
C10	0.0461 (13)	0.0571 (16)	0.0460 (15)	0.0197 (12)	−0.0060 (11)	−0.0060 (13)
C11	0.0375 (11)	0.0471 (13)	0.0278 (11)	−0.0064 (10)	−0.0034 (9)	−0.0041 (10)
C12	0.0338 (10)	0.0564 (15)	0.0358 (12)	−0.0054 (11)	−0.0087 (9)	0.0064 (11)
C13	0.0475 (14)	0.0614 (16)	0.0301 (12)	0.0111 (12)	0.0061 (10)	−0.0058 (12)
C14	0.0647 (17)	0.0607 (16)	0.0299 (13)	0.0123 (14)	−0.0096 (12)	0.0050 (12)
C15	0.0624 (16)	0.0310 (11)	0.0591 (19)	−0.0069 (12)	−0.0096 (14)	−0.0063 (12)
C16	0.0581 (16)	0.078 (2)	0.0430 (15)	0.0322 (17)	−0.0127 (13)	−0.0071 (15)

### Geometric parameters (Å, º)

**Table:**

N1—C5	1.467 (3)	C9—C14	1.519 (3)
N1—C6	1.487 (3)	C9—C16	1.534 (4)
N1—H1A	1.05 (4)	C9—H9	0.9800
N1—H1B	0.93 (3)	C10—C16	1.510 (4)
C2—C4	1.523 (3)	C10—H10A	0.9700
C2—C3	1.531 (3)	C10—H10B	0.9700
C2—C13	1.532 (3)	C11—C12	1.381 (3)
C2—C15	1.536 (3)	C11—H11	0.9300
C3—C6	1.515 (3)	C12—H12	0.9300
C3—C10	1.540 (3)	C13—H13A	0.9600
C3—H3	0.9800	C13—H13B	0.9600
C4—C5	1.393 (3)	C13—H13C	0.9600
C4—C11	1.395 (3)	C14—H14A	0.9600
C5—C7	1.386 (3)	C14—H14B	0.9600
C6—C9	1.518 (3)	C14—H14C	0.9600
C6—H6	0.9800	C15—H15A	0.9600
C7—C8	1.376 (3)	C15—H15B	0.9600
C7—H7	0.9300	C15—H15C	0.9600
C8—C12	1.378 (4)	C16—H16A	0.9700
C8—H8	0.9300	C16—H16B	0.9700
			
C5—N1—C6	113.23 (16)	C6—C9—H9	108.8
C5—N1—H1A	112 (2)	C14—C9—H9	108.8
C6—N1—H1A	110 (2)	C16—C9—H9	108.8
C5—N1—H1B	109.9 (18)	C16—C10—C3	105.4 (2)
C6—N1—H1B	109.6 (18)	C16—C10—H10A	110.7
H1A—N1—H1B	102 (3)	C3—C10—H10A	110.7
C4—C2—C3	107.70 (17)	C16—C10—H10B	110.7
C4—C2—C13	111.01 (19)	C3—C10—H10B	110.7
C3—C2—C13	108.59 (19)	H10A—C10—H10B	108.8
C4—C2—C15	108.32 (18)	C12—C11—C4	121.8 (2)
C3—C2—C15	112.4 (2)	C12—C11—H11	119.1
C13—C2—C15	108.9 (2)	C4—C11—H11	119.1
C6—C3—C2	112.69 (18)	C8—C12—C11	120.3 (2)
C6—C3—C10	103.27 (18)	C8—C12—H12	119.8
C2—C3—C10	119.81 (19)	C11—C12—H12	119.8
C6—C3—H3	106.8	C2—C13—H13A	109.5
C2—C3—H3	106.8	C2—C13—H13B	109.5
C10—C3—H3	106.8	H13A—C13—H13B	109.5
C5—C4—C11	116.5 (2)	C2—C13—H13C	109.5
C5—C4—C2	123.37 (19)	H13A—C13—H13C	109.5
C11—C4—C2	120.08 (19)	H13B—C13—H13C	109.5
C7—C5—C4	122.0 (2)	C9—C14—H14A	109.5
C7—C5—N1	116.36 (19)	C9—C14—H14B	109.5
C4—C5—N1	121.67 (18)	H14A—C14—H14B	109.5
N1—C6—C3	108.17 (16)	C9—C14—H14C	109.5
N1—C6—C9	115.09 (18)	H14A—C14—H14C	109.5
C3—C6—C9	105.22 (18)	H14B—C14—H14C	109.5
N1—C6—H6	109.4	C2—C15—H15A	109.5
C3—C6—H6	109.4	C2—C15—H15B	109.5
C9—C6—H6	109.4	H15A—C15—H15B	109.5
C8—C7—C5	120.1 (2)	C2—C15—H15C	109.5
C8—C7—H7	120.0	H15A—C15—H15C	109.5
C5—C7—H7	120.0	H15B—C15—H15C	109.5
C7—C8—C12	119.3 (2)	C10—C16—C9	108.7 (2)
C7—C8—H8	120.3	C10—C16—H16A	110.0
C12—C8—H8	120.3	C9—C16—H16A	110.0
C6—C9—C14	114.8 (2)	C10—C16—H16B	110.0
C6—C9—C16	101.64 (19)	C9—C16—H16B	110.0
C14—C9—C16	113.6 (2)	H16A—C16—H16B	108.3
			
C4—C2—C3—C6	48.4 (2)	C2—C3—C6—N1	−66.8 (2)
C13—C2—C3—C6	168.67 (19)	C10—C3—C6—N1	162.49 (19)
C15—C2—C3—C6	−70.8 (2)	C2—C3—C6—C9	169.71 (19)
C4—C2—C3—C10	170.1 (2)	C10—C3—C6—C9	39.0 (2)
C13—C2—C3—C10	−69.6 (3)	C4—C5—C7—C8	−1.1 (3)
C15—C2—C3—C10	50.9 (3)	N1—C5—C7—C8	177.7 (2)
C3—C2—C4—C5	−17.9 (3)	C5—C7—C8—C12	0.0 (4)
C13—C2—C4—C5	−136.7 (2)	N1—C6—C9—C14	79.9 (3)
C15—C2—C4—C5	103.8 (2)	C3—C6—C9—C14	−161.2 (2)
C3—C2—C4—C11	164.75 (19)	N1—C6—C9—C16	−157.0 (2)
C13—C2—C4—C11	46.0 (3)	C3—C6—C9—C16	−38.0 (2)
C15—C2—C4—C11	−73.5 (3)	C6—C3—C10—C16	−23.7 (3)
C11—C4—C5—C7	1.2 (3)	C2—C3—C10—C16	−150.0 (3)
C2—C4—C5—C7	−176.2 (2)	C5—C4—C11—C12	−0.1 (3)
C11—C4—C5—N1	−177.55 (19)	C2—C4—C11—C12	177.3 (2)
C2—C4—C5—N1	5.1 (3)	C7—C8—C12—C11	1.1 (4)
C6—N1—C5—C7	159.69 (19)	C4—C11—C12—C8	−1.0 (4)
C6—N1—C5—C4	−21.5 (3)	C3—C10—C16—C9	0.4 (3)
C5—N1—C6—C3	50.1 (2)	C6—C9—C16—C10	22.9 (3)
C5—N1—C6—C9	167.43 (18)	C14—C9—C16—C10	146.8 (3)

### Hydrogen-bond geometry (Å, º)

**Table:**

*D*—H···*A*	*D*—H	H···*A*	*D*···*A*	*D*—H···*A*
N1—H1*A*···Cl1	1.05 (4)	2.07 (4)	3.1201 (19)	173 (3)
N1—H1*B*···Cl1^i^	0.93 (3)	2.17 (3)	3.0943 (19)	174 (3)

Symmetry code: (i) *x*+1/2, −*y*+3/2, −*z*+1.
