# Supplementary figures and images for: Crystal structure of 3,9,9-trimethyl-2,3,3a,4,9,9a-hexa­hydro-1H-cyclo­penta[b]quinolin-4-ium chloride
Source: Acta Crystallogr E Crystallogr Commun. 2015 Jun 27;71(Pt 7):o525–6. doi: 10.1107/S2056989015011858 (PMC4518934; doi:10.1107/S2056989015011858)

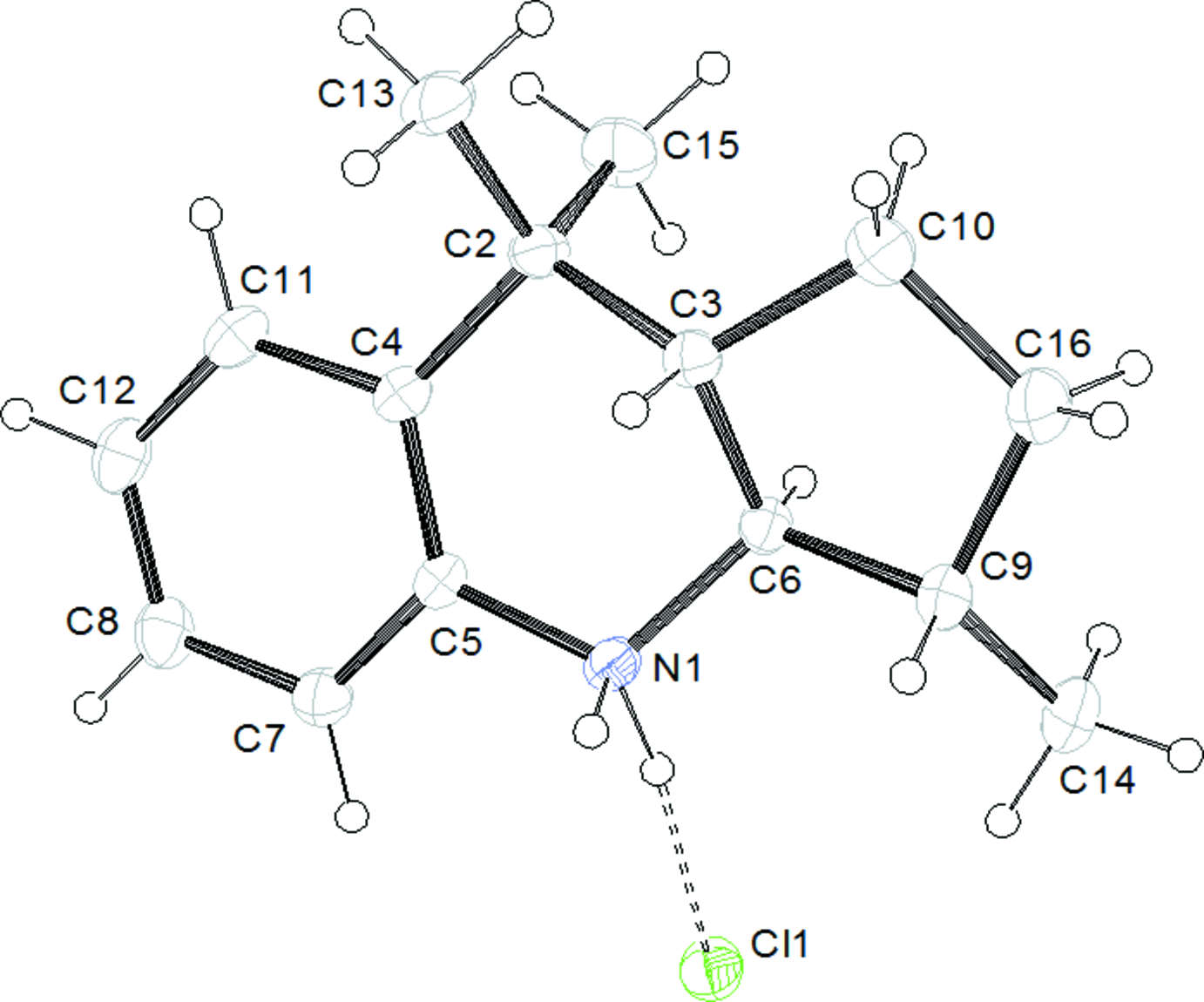

Supplement: Supplementary file 4 [file e-71-0o525-fig1.tif]

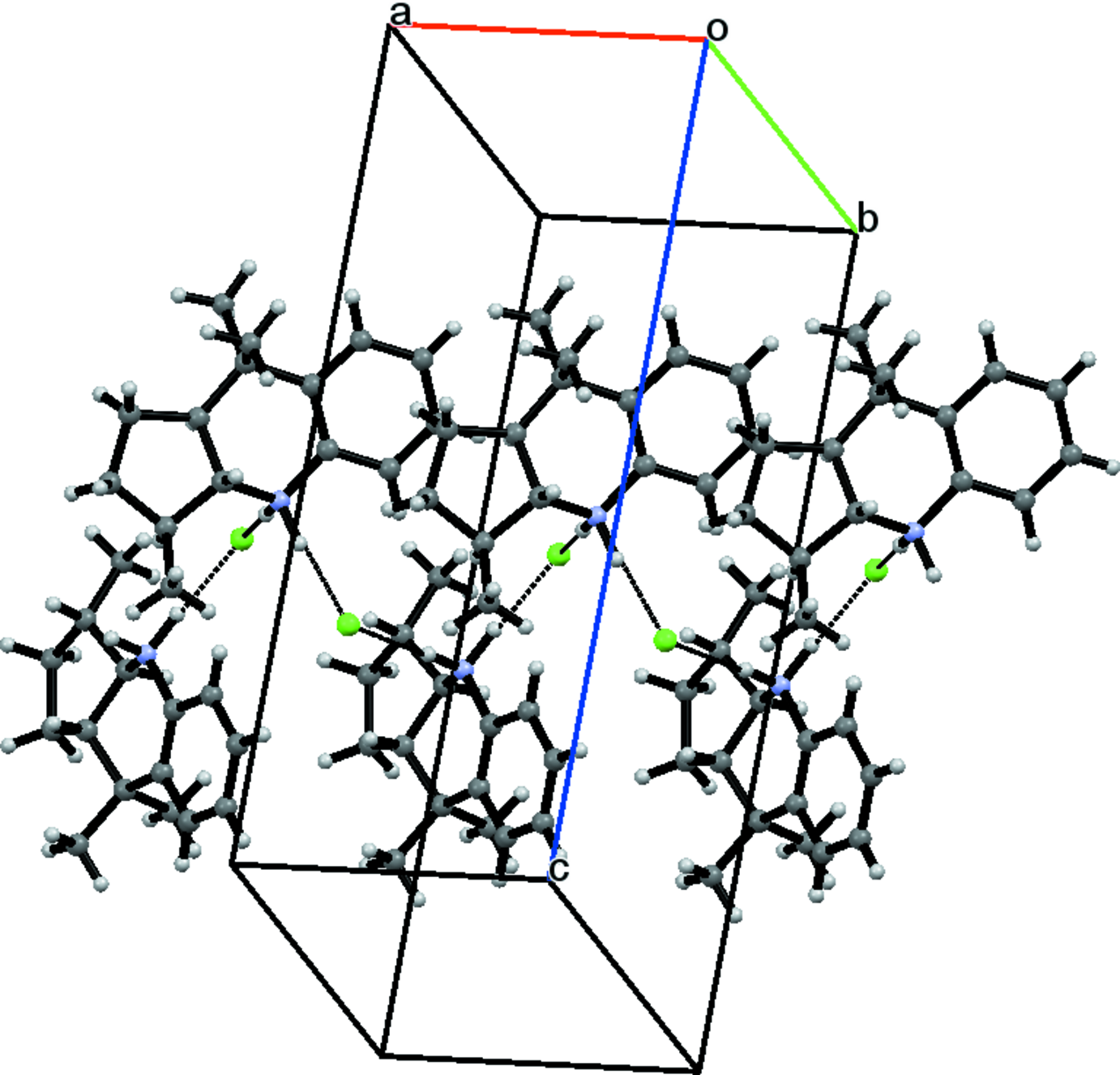

Supplement: Supplementary file 5 [file e-71-0o525-fig2.tif]
